# Wearables for Movement Analysis in Healthcare

**DOI:** 10.3390/s22103720

**Published:** 2022-05-13

**Authors:** Paolo Capodaglio, Veronica Cimolin

**Affiliations:** 1 Orthopaedic Rehabilitation Unit and Research Lab for Biomechanics, Rehabilitation and Ergonomics, Ospedale San Giuseppe, Istituto Auxologico Italiano, IRCCS, via Cadorna 90, 28824 Piancavallo di Oggebbio, Italy; 2 Department Surgical Sciences, Physical and Rehabilitation Medicine, University of Torino, 10126 Torino, Italy; 3 Department of Electronics, Information and Bioengineering, Politecnico di Milan, Piazza Leonardo da Vinci 32, 20133 Milan, Italy

Quantitative movement analysis is widely used in clinical practice and research to objectively and thoroughly investigate movement disorder. Conventionally, body segment kinematic and kinetic parameters are measured in gait laboratories, using marker-based optoelectronic systems, force plates, and electromyographic systems. Although movement analysis is considered accurate, the availability of specific laboratories, high costs, and dependency on trained users sometimes limit its use in clinical practice. A variety of available compact wearable sensors have allowed researchers and clinicians to pursue applications in which individuals are monitored in the home and community settings, in different fields, such as movement analysis. Wearable sensors may contribute to the out-patient implementation of quantitative movement analysis for clinical purposes, thereby reducing evaluation times and unobtrusively and continuously providing objective and quantifiable data on the patients’ capabilities.

We invited authors to submit their latest results in the field, either research articles or reviews articles, aimed at promoting novel wearable technology for movement analysis, methods for sensor signal processing, as well as on field experiences of their applications in healthcare. In total, 15 papers were accepted for publication in this Special Issue of *Sensors*, entitled “Wearables for Movement Analysis in Healthcare”. They are summarized in the subsequent paragraphs.

The papers could be divided into three main categories: methodological applications, clinical applications, and sport applications.

In terms of the methodological category, Zago et al. [1] estimated the gait parameters based on inertial sensors with machine learning techniques in healthy participants. Lueken et al. [2] presented a recently developed platform for a wireless body sensor network with customizable applications with a sensor setup for gait analysis during everyday life monitoring. Amitrano et al. [3] described a new wearable e-textile based system, named SWEET Sock, for the remote monitoring of biomedical signals and validated it by evaluating the agreement with an optoelectronic system for gait analysis on a set of free walk acquisitions.

In clinical applications, wearable sensors were used in several pathological states, such as stroke [4,5,6], obese [7,8], and elderly [9] patients; patients with lower limb amputation [10]; patients with Parkinson’s disease [4,11,12]; and patients hospitalized for knee joint rehabilitation [13]. In particular, wearable systems were used both to quantify the functional limitations of the patients, during several movements (gait [5,7,8,9], upper limb [6,11], time up and go test [10], and unconstraint activities at home [12]) and to evaluate their accuracy and precision in comparison with the gold standard [4]. These papers support the clinical usability of wearable technology for clinical movement assessment.

The applications in sport are more limited and they are focused on running and drop jump, forward sprint, and change in direction. Kim et al. [14] validated inertial measurement units (IMUs) for measuring ankle joint with a motion capture system during running in healthy individuals. Di Paolo et al. [15] quantified joint kinematics through a wearable sensor system in multidirectional high-speed complex movements after anterior cruciate ligament (ACL) injury, and validated it against a gold standard optoelectronic marker-based system. They demonstrated the use of wearable sensors as an alternative tool for motion capture system for assessing the performance and rehabilitation of athletes.

## Data Availability

Not applicable.
